# Assessment of vector control strategies based on mass *Aedes* (*Stegomyia*) mosquito trapping (AGOs traps) pilot study in a US-Mexico border region of South Texas

**DOI:** 10.1371/journal.pntd.0013665

**Published:** 2025-10-31

**Authors:** Jesús A. Aguilar-Durán, Josue Ramírez, Mario A. Rodríguez-Pérez, Christopher J. Vitek, Nadia A. Fernández-Santos, José Guillermo Estrada-Franco

**Affiliations:** 1 Instituto Politécnico Nacional, Centro de Biotecnología Genómica, Laboratorio de Biomedicina Molecular, Reynosa, Tamaulipas, México; 2 Health Department, City of Harlingen, Texas, United States of America; 3 Center for Vector-Borne, Zoonotic, and Emerging Diseases, University of Texas Rio Grande Valley, Edinburg, Texas, United States of America; 4 Department of Entomology, Texas A&M University, College Station, Texas, United States of America; Egerton University, KENYA

## Abstract

**Background:**

*Aedes* (*Stegomyia*) *aegypti* and *Ae.* (*Stegomyia*) *albopictus* mosquitoes are major vectors of diseases including dengue, Zika, chikungunya, and yellow fever. The excessive use of chemical insecticides has caused resistance in mosquito populations, along with negative environmental impacts and harm to non-target organisms. In this regard, mosquito control strategies, such as passive mass trapping interventions with autocidal gravid ovitraps (AGO) offer a promising alternative. Here we report the results of a pilot study evaluating a passive mass trapping treatment using AGOs against *Ae*. *aegypti* in the city of Harlingen, Texas, USA, during the peak mosquito season.

**Methodology:**

Three treatments were assessed on *Aedes* populations: AGO mass trapping, integrated vector management (IVM) consisting of source reduction together with larvicides and adulticides, and AGO + IVM. The study design included a control area with no treatments. Four neighborhoods were selected to evaluate the impact of treatments on *Ae*. *Aegypti* comparing female abundance between pre-treatment (10 weeks) and post-treatment (9 weeks) periods.

**Results:**

All treatments were effective in significantly reducing *Ae*. *aegypti* females. IVM treatment reduced the number of females per trap per week from 3.29 ± 0.24 to 2.41 ± 0.20 (33.7% reduction), AGO from 1.58 ± 0.17 to 0.25 ± 0.05 (85.2% reduction), and AGO + IVM from 1.49 ± 0.17 to 0.53 ± 0.08 (67.78% reduction), based on Henderson’s formula. We observed a non-significant increase in the control area (no treatment provided) in the mosquito populations, increasing from 2.94 ± 0.24 in the pretreatment period to 3.25 ± 0.28 of the post treatment period.

**Conclusion:**

Despite all treatments followed a reduction in mosquito populations, those that included AGO showed a greater decrease in post treatment populations than conventional control measures (IVM) alone. However, further studies with a larger number of replicates, conducted across different seasons and during peak abundance months are needed to fully assess their relative effectiveness for *Ae*. *aegypti* control. As this was a pilot study, these preliminary findings suggest that AGOs contribute to reducing *Ae*. *aegypti* populations and may serve as a complementary and useful tool in integrated vector management strategies. Nonetheless, further research is needed to verify and validate their effectiveness at larger operational scales.

## Author summary

*Aedes aegypti* mosquitoes are critically important vectors for global public health due to their primary role in the transmission of diseases such as dengue, Zika, chikungunya, and yellow fever, which affect millions of people worldwide, particularly in tropical and subtropical regions. Given the drawbacks of chemical insecticides used for vector mosquito control, including the development of insecticide-resistant mosquito populations, harm to non-target organisms, and environmental pollution, there has been increasing interest in evaluating alternative control strategies. We assessed the effectiveness of Autocidal Gravid Ovitraps (AGOs) in a passive mass trapping treatment to reduce *Ae*. *aegypti* populations. We compared this approach with conventional control measures (adulticides, larvicides and source reduction) as well as the combined effect of AGOs with conventional methods. All treatments successfully reduced mosquito densities; however, those that included AGOs were more effective than conventional control measures (IVM) alone. AGO treatment achieves the higher reduction with an 85% decrease in female *Ae*. *aegypti* mosquitoes. Our study aims to evaluate the potential of AGOs as a component of integrated vector management for reducing vector mosquitoes.

## Introduction

The Americas experienced the highest annual incidence of dengue disease since 1980 in 2024 [1]. In this context, two *Aedes* mosquito species, *Aedes* (*Stegomyia*) *aegypti* and *Aedes* (*Stegomyia*) *albopictus* play a central role in transmitting the disease, particularly in urban and peri-urban settings. These two species are also competent vectors for other arboviral diseases like Chikungunya and Zika [2]. Historically, arboviral diseases transmitted by *Aedes* mosquitoes have been present in the Southern United States since the 1700s through the 1940s [3]. During this period, outbreaks of dengue associated with *Ae. aegypti* mosquitoes prompted public health agencies to launch mosquito eradication campaigns [4]. However, after a 40-year period of relatively low arboviral disease incidence and the cessation of elimination campaigns, dengue outbreaks re-emerged in the 1980s, 1990s, and 2000s [5–8]. This resurgence was closely linked to the large populations of *Ae*. *aegypti* mosquitoes. Both *Ae*. *aegypti* and *Ae*. *albopictus*, known for their efficient dengue transmission, are prevalent year-round in the US-Mexico Texas border region [9].

Traditional control methods for *Aedes* mosquitoes often rely on chemical larvicides and adulticides, but their effectiveness is declining due to the widespread presence of resistance in these mosquito populations, particularly to pyrethroid insecticides [10]. To address this challenge, the US Centers for Disease Control and Prevention (CDC) in Puerto Rico has proposed the use of non-chemical vector control approaches as an alternative [11,12]. This approach involves the use of the Autocidal Gravid Ovitrap (AGO), which has shown effectiveness in field trials with promising results in Puerto Rico and Mexico [13,14].

In this study, we evaluate the effect of AGO traps on a passive mass trapping treatment on reducing *Ae. aegypti* densities in an urban area of Harlingen City in Cameron County of South Texas and compare their performance to conventional integrated vector management (IVM) strategies.

## Materials and methods

### Study sites selection

**Fig 1:**
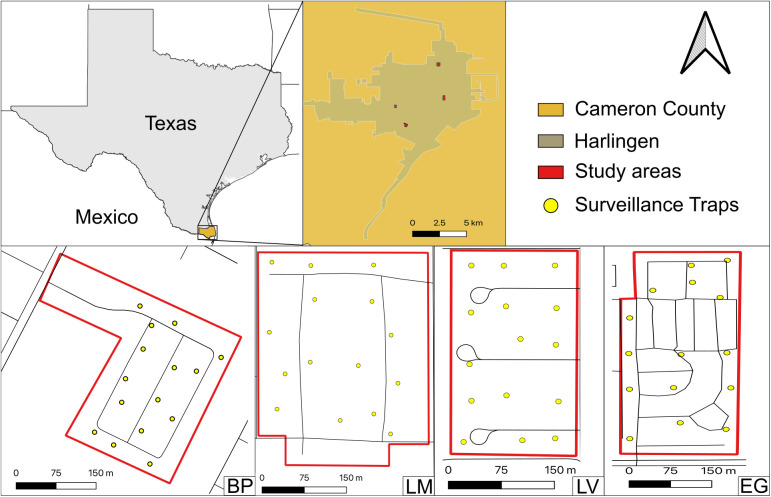
Map of Harlingen, TX showing the location of the four study neighborhoods. Location of the SAGOs indicated by yellow dots in urban neighborhoods of Harlingen, Texas. The four neighborhoods are depicted individually and delineated by red lines: BP: Bonita Park, LM: Le Moyne Gardens, LV: Los Vecinos, EG: Eastgate. Map made with QGIS 3.34.13 (https://qgis.org/en/site/) using TIGER/Line® shapefiles for roads, places, counties, and states from the U.S. Census Bureau (https://www.census.gov/geographies/mapping-files/time-series/geo/tiger-line-file.html).

We selected four neighborhoods within the City of Harlingen (26° 11’ 26.3“ N 97° 41’ 46.0” W) in Cameron County, Texas, United States, to conduct and assess the effectiveness of the proposed mosquito field experimental trials. All four neighborhoods were under the supervision of the Harlingen housing authority (Fig 1). The first one was Le Moyne Gardens (26°13’27.1” N 97°40’13.8” W), with 200 homes/apartments and 610 people in the whole complex (in 2019); the second one was Los Vecinos (26°11’05.7” N 97°42’39.3” W) with 150 homes/apartments and 248 inhabitants in the whole complex (in 2019); the third one was Bonita Park (26°10’00.8” N 97°42’05.4” W) with 120 homes/apartments and 367 inhabitants in the whole complex (in 2019). The fourth one was Eastgate (26°11’34.7” N 97°39’59.7” W) with 199 homes/apartment and a fluctuating population ranging 50–350 individuals depending on the season, with a peak population during December-February. The distance between neighborhoods ranged from approximately 2.1 km (Los Vecinos-Bonita Park) to 7.1 km (Le Moyne Gardens-Bonita Park), with an average inter-neighborhood distance of 4.52 km.

All selected neighborhoods share similar socioeconomic conditions, characterized by low house values and low-income levels with the widespread presence of *Ae*. *aegypti*, among other mosquito species. Vegetation and land cover were similar in all four neighborhoods and the main *Aedes* breeding sites were plastic water-holding containers, such as flowerpots. Housing density was relatively similar across most neighborhoods, with Bonita Park at 1.50, Le Moyne Gardens at 1.73 and Los Vecinos at 1.88 houses per 1,000 m^2^, respectively. In contrast, Eastgate showed a higher density of 2.68 houses per 1,000 m^2^.

Prior to beginning of the study, at least two townhall meetings were held in each of the four selected neighborhoods to motivate, inform and encourage community participation. These meetings included educational talks, informational pamphlets on mosquito prevention, and short videos as well as information of the objectives of the study and its benefits from preventing mosquito bites and disease.

### Experimental design

Three different treatments were evaluated using a repeated-measures design: the placement of three AGOs; an IVM approach that included source reduction, larviciding with Mosquito Dunks (*Bacillus thuringiensis israelensis*) and adulticiding with Mosquitomist 1.5 ULV (Chlorpyrifos 19.36%); and a combination of both treatments (AGO + IVM). A control area where no intervention was provided was included. The effectiveness of these treatments was evaluated based on the reduction of *Ae*. *aegypti* female populations by comparing the temporal changes in female densities across the four areas. Each of the four neighborhoods was randomly assigned a treatment or designated as the control. The experimental design included a 10-week pre-treatment period from August to October 2019 and a 9-week post- treatment period from October to December 2019.

To evaluate the efficacy of the treatments, approximately 100 houses per area were selected for each neighborhood. Bonita Park was designated as the control area, Le Moyne Gardens (116 houses selected) received the AGO trap treatment, Eastgate (105 houses selected) received the IVM treatment, and Los Vecinos (109 houses selected) received the combined AGO + IVM treatment.

Autocidal gravid ovitraps (AGOs) are passive mosquito traps designed to attract and capture gravid female *Ae*. *aegypti* seeking oviposition sites. Each trap consisted of a 19-liter black plastic bucket containing a hay infusion as an attractant, with a sticky surface at the entrance to capture the mosquitoes [11]. AGOs were deployed in large numbers as part of the treatments for mosquito population suppression, while sentinel autocidal gravid ovitraps (SAGOs) of identical design were kept as fixed location solely as monitoring tools and evaluation.

In order to monitor the number of *Ae*. *aegypti* females, sixty SAGOs were deployed, with fifteen placed within each of the four neighborhoods. To ensure even distribution, the traps were positioned in the front yards of randomly selected houses, maintaining a maximum distance of 100 meters between each trap (Fig 1). The monitoring of *Ae. aegypti* females in the pre-treatment period took place weekly from August 11 to October 13, 2022 (10 weeks).

Following the pre-treatment period, the assigned treatments (AGO, IVM, AGO+IVM) were implemented. The AGO treatment involved placing a total of three traps distributed in the front and backyards of most households within the area, except for households with a SAGO trap, where only two additional AGOs were deployed, dispersed at front and backyards. These AGOs remained at the houses the entire 9-week post-treatment period. The IVM treatment which included source reduction, larvicide, and adulticide, was carried out as a single application at the end of the pre-treatment period (10th week). Source reduction measures include the removal of small artificial containers, with homeowner or resident authorization. Larvicides (Mosquito Dunks containing *Bacillus thuringiensis israelensis*) were applied to other water-holding containers not used for animal or human consumption, including flowerpots and retention ponds. The adulticide used was Mosquitomist 1.5 ULV (Chlorpyrifos 19.36%), and applied around the perimeter of the selected houses in Eastgate and Los Vecinos. The control area maintained the existing SAGO traps without treatments.

The weekly post-treatment sampling period occurred from October 20 to December 15, 2019 (9 weeks). Every two months, AGOs received preventive maintenance that included replacing sticky glue boards, infusions of grass, and removal of external dirt. All captured mosquitoes in SAGOs were visually identified to species and sexed and their numbers used to build the mosquito database (S1 Table).

### Weather

In order to account for weather-related variations in *Ae*. *aegypti* females, we collected daily meteorological data (temperature, rainfall, and relative humidity) from the nearest airport weather station at Valley International Airport in Harlingen, from August 04 to December 22, 2019.

### Statistical analysis

To investigate the presence of a significant interaction between captured *Ae*. *aegypti* females in SAGOs, traps, and sampling weeks (S1 Table). An analysis of covariance (ANCOVA) was conducted using the MIXED procedure with a first-order autoregressive structure variance [15]. This ANCOVA application facilitated the control of potential covariates that could influence mosquito abundance. This approach, crucial for repeated-measures studies, allowed a more robust evaluation of treatment effects and a more reliable interpretation of the results [16]. The experimental unit in this repeated-measures study was the SAGOs, with the response variable being the number of females caught, and the random factors were the location and number of traps. A generalized linear mixed model (GLMM) was employed to assess the impact of weather conditions, temperature, rainfall, and relative humidity, on mosquito abundance.

Furthermore, to assess the impact of the treatments (AGO, IVM, AGO + IVM) on the abundance of *Ae*. *aegypti*, the mean number of *Ae*. *aegypti* females per SAGO per week were compared between the 10-week pre-treatment period and the 9-week post-treatment period. The four treatments and the time (pre or post) were considered as predictors and class variables in the multivariate negative binomial regression model.

The statistical model’s goodness of fit was assessed using Pearson’s chi-square test for degrees of freedom (χ^2^/df). Results lower than two were considered indicative of a well-fitting model.

Differences between treatments and time were examined using the GLIMMIX procedure [17], which is designed to assess the impact of categorical, discrete, or continuous variables on a count response variable, regardless of the probability distribution, Additionally, comparisons between treatments and control areas were determined as a relative reduction in female *Ae*. *aegypti* using a modified version of Henderson’s formula [18] as shown:


$$\left(\%\text{Reduction}=\;\left(\text{1}-\left(\frac{{\text{Treatment}}_{t}}{{\text{Treatment}}_{Pre}}/\frac{{\text{Control}}_{t}}{{\text{Control}}_{Pre}}\right)\right)*\text{1}\text{0}\text{0}\%\right)$$

where subscripts represent the mean number of females at time *t*, the pre-treatment mean and control mean. All statistical analyses were conducted using SAS OnDemand for Academics [19].

## Results

A total of 2,592 female mosquitoes captured during this 19-week study, with *Ae*. *aegypti* representing 88.8% (2,303) and *Ae*. *albopictus* representing 11.2% (289). Due to the low number of *Ae*. *albopictus*, data for this species were excluded from further analysis.

At the beginning of the study, 100% coverage was achieved with the SAGOs. However, some SAGOs were later vandalized, removed, or moved to another collecting site, resulting in lower coverage. In Le Moyne Gardens (AGO treatment), 110 out of 116 houses (94.8%) retained the traps. In Eastgate, all 105 houses (100%) received the IVM treatment, while in Los Vecinos, 99 out of 109 houses (85.8%) retained the AGO and received the IVM treatment.

Regardless of the treatments, the covariance analysis results (Cov = 0.02386; *χ* = 0.22, *df* = 1, *p* = 0.6402) show no significant interaction in the number of captured *Ae. aegypti* between traps or weeks. Therefore, the female abundance was independent between treatments and weeks.

The average number of *Ae*. *aegypti* females per trap per week in Bonita Park, the control area that did not receive any treatment, ranged from 2.94 ± 0.24 before treatments to 3.25 ± 0.28 after treatments. In Eastgate (IVM treatment), the average number on females collected decreased from 3.29 ± 0.24 to 2.41 ± 0.20. Le Moyne Gardens (AGO area) saw a decreased from 1.58 ± 0.17 to 0.25 ± 0.05; and Los Vecinos (AGO + IVM area) decreased from 1.49 ± 0.17 to 0.53 ± 0.08. A summary of female *Ae*. *aegypti* abundance and the corresponding percentage reduction across treatment areas is showed in Table 1.

**Table 1:** Average number of female *Ae*. *aegypti* in SAGOs traps per trap per week before and after treatments.

Area	Treatment	Pre-treatment (Mean ± SE)	Post-treatment (Mean ± SE)	% Reduction^a^	Statistical metrics^b^
Bonita Park	Control	2.94 ± 0.24	3.25 ± 0.28	—	*F*_(1, 283)_ = 0.70, *P *= 0.4046
Eastgate	IVM	3.29 ± 0.24	2.41 ± 0.20	33.7%	*F*_(1, 283)_ = 7.62, *P *= 0.0062
Le Moyne Gardens	AGO	1.58 ± 0.17	0.25 ± 0.05	85.2%	*F*_(1, 283)_ = 59.72, *P *< 0.0001
Los Vecinos	AGO + IVM	1.49 ± 0.17	0.53 ± 0.08	67.78%	*F*_(1, 283)_ = 25.72, *P *< 0.0001

^a^Percentage reduction was calculated using a modified version of Henderson’s formula.

^b^Statistical comparisons were performed using a Generalized Linear Mixed Model (PROC GLIMMIX).

**Fig 2:**
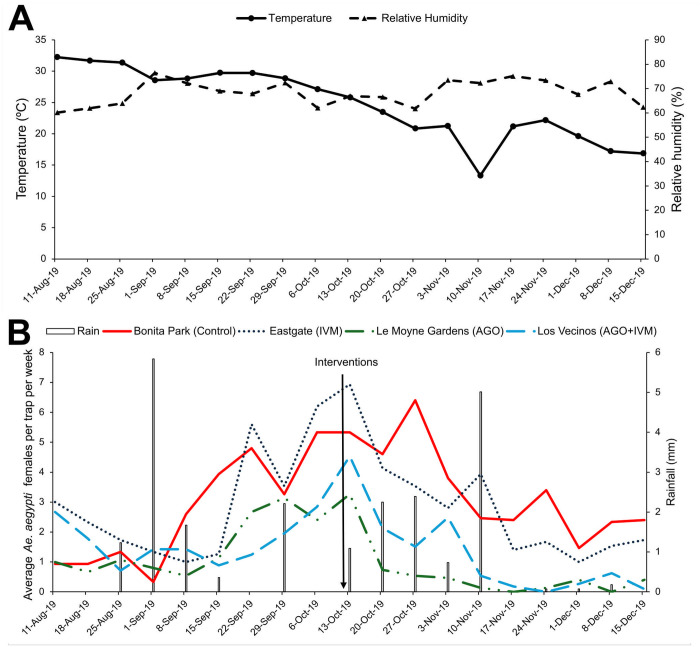
Weather conditions and variation in the numbers of female *Ae. aegypti* captured in SAGO traps. **(A)** Weekly average weather conditions during the 19-weeks study. **(B)** Average number of *Ae*. *aegypti* females per SAGO per trap per week. Bonita Park (control neighborhood), Le Moyne Gardens (AGO neighborhood), Eastgate (IVM neighborhood), and Los Vecinos (AGO + IVM neighborhood).

The GLMM analysis revealed significant effects of temperature (GLIMMIX: *F*_(1, 1133)_ = 18.10, *P* < 0.0001) and relative humidity (GLIMMIX: *F*_(1, 1133)_ = 18.12, *P* < 0.0001) on the number of *Ae*. *aegypti* females per trap per week (Fig 2A), while rainfall had no significant impact (GLIMMIX: *F*_(1, 1133)_ = 3.70, *P* = 0.0545). A general decline in mosquito abundance was observed towards the end of the study, most likely caused by the decline in temperature during the last weeks of the study (Fig 2B).

All three treatments significantly reduced *Ae. aegypti* female densities compared to control. The IVM treatment achieve a 33.7% reduction in the number of females (GLIMMIX: *F*_(1, 283)_ = 7.62, **P* *= 0.0062). The AGO treatment achieved the highest reduction at 85.2% (GLIMMIX: *F*_(1, 283)_ = 59.72, **P* *< 0.0001). The combination of both treatments (AGO + IVM) resulted in a 67.78% reduction (GLIMMIX: *F*_(1, 283)_ = 25.72, **P* *< 0.0001). As expected, the control area showed no significant change in female density, with any variation between pre- and post-treatment trapping likely due to seasonal population dynamics (GLIMMIX: *F*_(1, 283)_ = 0.70, **P* *= 0.4046).

## Discussion

We found that all the treatments (IVM, AGO, and AGO + IVM) significantly reduced the abundance of female *Ae*. *aegypti* when comparing the pre-treatment to post-treatment numbers, within each treatment area. As expected, the control site did not show any significant changes in mosquito densities.

Among all treatments, AGOs showed the highest reduction (85.2%) compared to the control area at the end of the 20-week study period. These findings are consistent with a similar field study in northeastern Mexico, where three similar treatments (AGOs, IVM, and AGO + IVM) were evaluated. In northeastern Mexico, the AGO treatment achieved the most significant reduction in female mosquito densities, with a 47% decrease [17], which is lower than that of 85.2% of the present study in Harlingen, TX.

The IVM treatment resulted in a 33.7% reduction in female mosquito densities, showing a significant decrease following the application of the treatment (Fig 2B). This suggests that Harlingen populations of *Ae*. *aegypti* are still somewhat susceptible to the insecticide and larvicide used. To our knowledge, there are no reports of resistance to *Bacillus thuringiensis israelensis* (Bti) [20] and Chlorpyrifos [21,22] in mosquito populations from South Texas. Therefore, insecticide resistance is unlikely to have impacted our study.

Surprisingly, the combination of IVM and AGO did not enhance the reduction of *Ae*. *aegypti* abundance compared to AGO alone, although it was more effective than IVM alone. The combination of AGO and IVM resulted in a smaller decrease, with a 67.78% reduction compared to the 85.2% reduction for AGO alone. We hypothesize that this effect may be due to differences in coverage and availability of resting and breeding sites. Although both areas achieved 100% coverage at the study initiation, the final coverage was 94.8% in Le Moyne Gardens (AGO area) compared to 85.8% in Los Vecinos (AGO + IVM area). Similarly, in a field evaluation of AGOs [14], the most significant reduction in mosquito densities was observed when coverage with AGOs was highest. Therefore, this ~10% difference in coverage may have influenced the level of success in control efforts. Furthermore, Le Moyne Gardens was more isolated from neighboring communities than Los Vecinos, which is located near the city center, therefore it could have a higher number of potential breeding sites. In addition, mosquito migration from surrounding areas may have influenced mosquito densities. Our data suggests that there may be a critical density of AGOs needed for effective control effort, although this critical density may vary based on geographic and environmental factors.

The drawbacks associated with using chemical insecticides, such as the development of resistance mosquito populations, environmental pollution, and harmful effects on non-target organisms, have led to increased interest in researching alternative methods for controlling vector mosquito populations [23,24]. Among these, passive mass trapping treatments carried out with AGOs stand out as a promising tool with successful results in Puerto Rico, decreasing the densities of female *Ae*. *aegypti* and reducing arbovirus transmission in field studies [12–14]. In the USA, preliminary studies also show the potential of AGOs in controlling *Ae*. *aegypti* and *Ae*. *albopictus* when used alongside conventional methods [25,26].

Since mosquitoes are trapped and killed on the sticky glue board of the trap, AGOs can be used in areas where chemical resistance is present [11]. Additionally, AGOs can serve as an effective surveillance tool for monitoring populations of *Ae*. *aegypti*, *Ae*. *albopictus* as well as another vector species *Culex quinquefasciatus* [27–30], reinforcing their role as a key component of integrated vector control strategies.

Weather conditions, such as temperature and humidity, significantly influence the life cycle, dispersal patterns, and physiology of *Aedes* mosquitoes [31–33]. Among these, temperature is one of the most significant abiotic factors on mosquito development. Low temperatures (<10 **°**C) can hinder mosquito movement and larval development, while higher temperatures may accelerate their life cycle but decrease hatching rates and hinder the development of adults [31]. Rainfall also plays a crucial role by increasing mosquito abundance and breeding opportunities; however, excessive precipitation can be detrimental [34,35]. In this study, temperature affected the mean number of *Ae*. *aegypti* females across all neighborhoods. At the beginning of the study, high temperatures exceeding 30**°**C were associated with low mosquito densities, while mosquito numbers increased when temperatures dropped slightly to 25–28 **°**C. In the final weeks, temperatures decreased below 20 **°**C, corresponding with a decrease in the number of *Ae*. *aegypti* females in all areas.

These findings align with studies conducted in Puerto Rico using AGOS, where average temperature and relative humidity influenced female *Ae*. *aegypti* densities. Additionally, rainfall was found to impact the average number of females captured per trap per week in these studies [11–14]. Contrary to their findings, rainfall was not a significant covariate, which may be attributed to the fewer rainy days in southern Texas (approximately 25 d during the study period) compared to the extended rainy season in Puerto Rico.

These results were part of a binational project in northeastern Mexico and southwestern United States, aimed in evaluating the impact of AGOs in decreasing female *Ae*. *aegypti* populations in two Mexico-USA border cities: Reynosa, Tamaulipas, Mexico and Harlingen, Texas, in the United States. Results from Reynosa, have been previously published [17], providing insights about the effectiveness of passive mass trapping treatments with AGOS in low-income disadvantaged neighborhoods with significant mosquito infestation problems, expanding upon findings from similar studies in Puerto Rico.

While both study areas shared similar socio-economic conditions, a key difference was in housing conditions: Reynosa’s neighborhoods had higher levels of crowding conditions compared to the larger and more spacious houses in Harlingen. However, these differences did not impact the efficacy of AGOS in decreasing mosquito densities in both cities.

By encouraging cooperation between the two countries, this project not only takes advantage of shared resources and expertise, but it also underscores the importance of coordinated binational efforts to address and advocate the risks of vector-borne diseases across borders.

One of the study limitations was having just a single replicate of each treatment, which precludes the possibility of drawing firm conclusions regarding the treatments impact. In addition, because the study was conducted in an urban setting, we were unable to completely isolate the evaluated areas. Consequently, the possibility that mosquito migration from non-selected areas could have affected the results cannot be ruled out.

Pre-treatment differences in *Ae*. *aegypti* densities across neighborhoods also suggests local environmental and ecological variations because the availability of water-holding containers, vegetation and human behavior, which can significantly influence mosquito breeding and survival. As a result, some neighborhoods might have been more favorable for *Ae*. *aegypti* proliferation, which it would have influenced the relative impact of the treatments [36–38].

Fluctuations in human populations may also influence mosquito population dynamics independently of treatments. We observed such point especially in Eastgate (IVM treatment) where the resident number varied considerably (from 50 to 600 inhabitants). Higher human population is often associated with increased availability of larval breeding sites and blood meal sources, potentially increasing *Ae*. *aegypti* densities. Moreover, residential density was higher in Eastgate compared to the rest of neighborhoods. This would have contributed to mosquito abundance by facilitating mosquito dispersal and host-seeking efficiency [37,39].

Conducting the treatments in October, when temperatures start to drop, presented additional problems as mosquito populations decline with colder temperatures [32,40]. This may have masked the true impact of treatments on *Ae*. *aegypti* populations. Performing the treatments during the months with the highest abundance (September-November) [17,41] in the region, along with increasing the number of replicates in follow up studies, it could provide more precise insights into the impact of treatments on mosquito densities. In addition, conducting treatment efforts during the first peak of mosquito abundance in the spring (April – June), may impact population densities later in the season as well.

This is the first report on the impact of passive mass trapping with AGOs as a control measure against *Ae*. *aegypti*, in Harlingen, Texas, US. Our results showed that AGOs effectively reduce abundance of female *Ae*. *aegypti*, suggesting their potential value as a complementary strategy within integrated vector control programs. While the findings are promising, their interpretation should be conservative, considering the pilot-scale nature of this study. Further research on a larger scale and across different seasons is needed to provide a better understanding of the long-term effectiveness and usefulness of these traps.

## Supporting information

S1 Table *Aedes aegypti* database from SAGO traps. (XLSX)
